# Some Atomic Absorption Spectrometric Applications to Clinical-Biomedical Trace Metal Analyses

**DOI:** 10.6028/jres.093.066

**Published:** 1988-06-01

**Authors:** Eleanor Berman

**Affiliations:** Division of Biochemistry, Cook County Hospital, Chicago, IL 60612

Following the introduction of atomic absorption spectrometric (AAS) instrumentation about 30 years ago, many specific and sensitive methods for determining trace metal concentrations in biological materials were developed. Yesterday’s rare and esoteric investigations are today’s routine clinical analyses. Levels of essential and toxic metals can be determined with relative ease for diagnostic purposes and following response to treatment. There is greater understanding of the chemistry and biochemistry of trace metals in health and disease as a consequence.

## The Challenges

Atomic absorption spectrometric analyses of the trace metal content of biological materials are challenging. These materials are complex, containing components that can generate nonspecific molecular absorption signals which may bias absorption measurements of the trace metals of interest. Sodium, and to a lesser extent potassium and protein, represent the major interferences.

Biological materials usually require pretreatment before instrumental analyses: the extent necessary depends upon the material itself as well as the concentration of the analyte. Tissues must be solubilized while blood and urine may require the removal of the proteins.

The sensitivity or detection limit capability of the instrument is an important factor. Oftentimes trace analytes in biological tissues and fluids are below the detection limits of the analytical instrumentation and a preconcentration is required.

Unknowns and standards should be similar to obtain valid instrumental comparisons. A blank or zero concentration standard is included with each group of assays. Control materials should be determined concurrently to assure the quality of the assay. There are standard reference materials available for most trace metals.

## Contamination

Many of the trace metals of clinical interest are common contaminants in the laboratory environment and in areas where samples are collected for analysis. Maintenance of a clean environment from the point of sample collection to the release of analytical findings is a continuous challenge for the trace metal analyst in a clinical laboratory. Rigorous, but practical, measures must be instituted and maintained to ensure that sample collection vessels, measuring devices, labware in general, water, and reagents are essentially free from trace metal contaminants. For example, determining trace metal residues on freshly cleaned labware and setting limits of acceptance can prove quite effective in maintaining the quality of labware and reagents, and ultimately, the quality of the analytical measurements.

Since the skin and clothing of subjects being investigated for exposure to toxic metals such as lead, cadmium, and mercury are often liberally dusted with the metals of interest, it is prudent for the laboratory to request, periodically, that sponges used in cleaning the venipuncture sites be submitted along with the blood specimens sent for analysis. If venipuncture sites are not cleaned adequately, sufficient contamination can be introduced into the blood samples to yield erroneously elevated levels.

To illustrate: the amount of lead removed by alcohol sponges from the venipuncture sites of 20 battery workers was found to range from 0.5 to 14.9 mg. Four to five separate sponges were required to free the skin area from lead contaminants. Initial lead content of the brand of alcohol sponges used in the study was 30 to 60 μg.

## Lead, Cadmium, and Thallium

The many procedures described for determining the toxic metals lead, cadmium, and thallium vary in complexity from simple dilution with a surfactant to precipitation of proteins by nitric or tri-chloroacetic acids to chelation-solvent extraction techniques at different pH ranges [1]. In the latter case, we found a pH of 5.5 to 6.5 to be optimal.

Capability of the instrumentation available, as well as the population being investigated govern the choice of method adopted. Both flame and electrothermal atomization techniques can be employed. For flame methods larger sample size is needed while electrothermal atomization is usually more than an order of magnitude more sensitive. All techniques have their pitfalls. For example, versenate (EDTA) blocks the solvent extraction of lead as a dithiocarbamate [2]. EDTA is a stronger chelator of lead than is dithiocarbamate. Furthermore, the lead-EDTA complex is water soluble and is not extracted by an organic solvent. The addition of calcium can eliminate the versenate interference with the chelation-extraction of lead [3]. However, recoveries tend to be variable. The acid precipitation methods [4] for lead analysis are not influenced by versenate; however, versenate does not interfere with the chelation-extraction of cadmium, thallium, or mercury as dithiocarbamates.

Both techniques, chelation-extraction and acid precipitation, yield comparable results when blood samples from asymptomatic children and adults or NBS porcine blood lead controls are analyzed. However, higher values are obtained on blood samples drawn from symptomatic subjects [5]. Table 1 lists a comparison between the two methods.

This pattern follows the subjects throughout treatment and subsequently. Since agreement between the two procedures was good when applied to control materials and blood samples from asymptomatic subjects, incidental error can be ruled out. It is possible, perhaps, that the low molecular weight protein described by Raghavan and Gonick [6] may be a factor in producing the discrepancies observed between the two methods when applied to blood lead analyses of symptomatic individuals. This protein, occurring in the red cells of lead exposed subjects, was found to bind considerable lead. Quite possibly, this boundlead is precipitated along with the blood proteins.

## Mercury

Determination of mercury in a clinical laboratory presents special challenges. Because of the volatility of elemental mercury and some of its compounds even at ambient temperatures, precautions must be taken to prevent losses of the element during the analytical process.

For obvious reasons mercury analyses should not be performed in a room containing a Van Slyke or comparable type apparatus. Furthermore, wearing of certain cosmetics, for example, eye shadows, by technical personnel doing these analyses should be prohibited. Many such preparations contain mercury salts.

Some current analytical methods utilize an adaptation of the cold-vapor technique wherein mercury is reduced to the elemental state and swept from solution by a stream of inert gas and into an absorption tube in an atomic absorption spectrometer. Mercury vapor, so measured, is essentially free from interferences due to matrix constituents [7,8].

This technique proves to be a bit awkward in the usual high volume clinical laboratory; however, we find chelation-extraction less cumbersome. Mercury in solution is chelated at pH 3–4 by ammonium pyrollidine dithiocarbamate and extracted into methyl isobutyl ketone. Standards and unknowns are compared in a graphite furnace programmed to dry and char at 75 °C. Background correction is necessary.

## Therapeutic and Essential Elements

Determination of serum levels of the therapeutic metal lithium and the essential elements magnesium, copper, zinc, and iron can prove to be life saving guides to the immediate therapy indicated. Sample preparation involves only dilution or protein precipitation since levels of the metals are in the μg/mL range compared to mg/mL quantities for the toxic elements.

Lithium and copper are distributed equally between cells and serum. However, red cells contain more magnesium, zinc, and iron than does serum. To assure analytical accuracy, hemolysis should be avoided. Also, cells and sera must be separated shortly after sample collection.

Since aqueous solutions leach magnesium from glass containers, materials and reagents meant for magnesium analysis should be stored in plastic containers washed to reduce trace metal content.

Specimens for zinc analysis are best collected and stored in washed plastic containers to avoid contamination by the zinc present in rubber stoppers of the usual evacuated tubes.

Both flame and electrothermal atomization techniques can be applied to the analyses of these metals. Flame atomization is more practical for routine clinical determinations of lithium, magnesium, and zinc. Electrothermal atomization is preferred for copper and iron analyses. Background correction is essential for electrothermal atomization AAS.

## Figures and Tables

**Table 1:** Comparison of blood lead values obtained by chelation-extraction and nitric acid precipitation methods

Subject	Status	Lead level μg% Chelation-extraction	Nitric acid
child	asymptomatic	36	34
child	asymptomatic	29	29
child	asymptomatic	58	58
child	symptomatic	56	34
child	symptomatic	56	29
child	symptomatic	143	93
adult	asymptomatic	24	23
adult	asymptomatic	45	44
adult	asymptomatic	57	58
adult	symptomatic	69	49
adult	symptomatic	57	44
adult	symptomatic	67	48
